# Interfacility Helicopter Ambulance Transport of Neurosurgical Patients: Observations, Utilization, and Outcomes from a Quaternary Level Care Hospital

**DOI:** 10.1371/journal.pone.0026216

**Published:** 2011-10-12

**Authors:** Brian P. Walcott, Jean-Valery Coumans, Matthew K. Mian, Brian V. Nahed, Kristopher T. Kahle

**Affiliations:** Department of Neurosurgery, Massachusetts General Hospital and Harvard Medical School, Boston, Massachusetts, United States of America; City of Hope, United States of America

## Abstract

**Background:**

The clinical benefit of helicopter transport over ground transportation for interfacility transport is unproven. We sought to determine actual practice patterns, utilization, and outcomes of patients undergoing interfacility transport for neurosurgical conditions.

**Methodology/Principal Findings:**

We retrospectively examined all interfacility helicopter transfers to a single trauma center during 2008. We restricted our analysis to those transfers leading either to admission to the neurosurgical service or to formal consultation upon arrival. Major exclusion criteria included transport from the scene, death during transport, and transport to any area of the hospital other than the emergency department. The primary outcome was time interval to invasive intervention. Secondary outcomes were estimated ground transportation times from the referring hospital, admitting disposition, and discharge disposition. Of 526 candidate interfacility helicopter transfers to our emergency department in 2008, we identified 167 meeting study criteria. Seventy-five (45%) of these patients underwent neurosurgical intervention. The median time to neurosurgical intervention ranged from 1.0 to 117.8 hours, varying depending on the diagnosis. For 101 (60%) of the patients, estimated driving time from the referring institution was less than one hour. Four patients (2%) expired in the emergency department, and 34 patients (20%) were admitted to a non-ICU setting. Six patients were discharged home within 24 hours. For those admitted, in-hospital mortality was 28%.

**Conclusions/Significance:**

Many patients undergoing interfacility transfer for neurosurgical evaluation are inappropriately triaged to helicopter transport, as evidenced by actual times to intervention at the accepting institution and estimated ground transportation times from the referring institution. In a time when there is growing interest in health care cost containment, practitioners must exercise discretion in the selection of patients for air ambulance transport—particularly when it may not bear influence on clinical outcome. Neurosurgical evaluation via telemedicine may be one strategy for improving air transport triage.

## Introduction

The Emergency Medical Treatment and Active Labor Act effectively requires Level I trauma centers to accept all transfers for a higher level of care if hospital capacity exists.[1] As such, Level I trauma centers typically provide tertiary and quaternary specialty coverage to a wide geographic area.[2] It is common for patients to be transferred to academic medical centers from other hospitals for neurosurgical care.[3] Most of these patients are transported either via ground or helicopter ambulance.

Though various state-specific and Centers for Disease Control issued guidelines exist regarding the triage of trauma patients to helicopter from the scene of injury [4], the decision to transfer a patient from a referring hospital via air ambulance is left to the discretion of the health care practitioner. In general, it is assumed that interfacility transfer via helicopter reduces transfer time relative to ground ambulance, allowing for more rapid intervention at the accepting institution.

Whether air transport times are actually faster and whether such reductions in transfer duration confer a clinical benefit are debated, and there has been no randomized, controlled trial comparing outcomes after transfer via helicopter versus ground ambulance. A study of 1,234 critical patients transported between facilities by helicopter demonstrated no improvement in outcomes over those transported by ground [5], but another study reported decreased mortality among patients transferred by air.[6] As the incidence of helicopter transport increases, it is essential to identify patients that may benefit most from this mode of transportation, mindful of its unique cost profile.[7]–[8]

We aimed to identify practice patterns in helicopter transport related to resource utilization and clinical outcomes among patients undergoing interfacility transfer for neurosurgical indications. We hypothesize that many of these patients are inappropriately triaged to helicopter transport, and we use actual times to neurosurgical intervention and estimated ground transportation times from the referring hospital to substantiate this.

## Methods

### Objectives

The primary outcome assessed was time to invasive neurosurgical intervention. Secondary outcomes were estimated ground transportation times from the referring hospital, admitting disposition, and discharge disposition.

### Participants

We identified 526 helicopter transports from 91,435 emergency department patient visits at a single level I adult and pediatric trauma center (certified by the American College of Surgeons) in Boston, MA USA during 2008. Study inclusion criteria consisted of interfacility transfer from an emergency department via helicopter followed by either neurosurgical consultation or admission upon arrival. In addition, we required the primary diagnosis to be neurosurgical. Major exclusion criteria included transport from the scene, death during transport, and transport to any area of the hospital other than the emergency department. All subjects had electronic medical records allowing for exact determination of study outcome measures.

### Investigations Undertaken

Data from the electronic medical record were collected for cases meeting study criteria, including patient demographics, referring hospital location, neurosurgical diagnosis, admitting disposition, discharge disposition, time to neurosurgical intervention (if any; time was defined as the interval between arrival in the emergency department and arrival in the operating room), and length of hospital stay. We estimated driving times with Google Maps software (Google Inc. Mountain View, CA USA). We did not attempt to account for traffic or weather conditions at the time of transport. This web-based software is free and available to the public domain. Several medical studies have also reported using this geocoding software to analyze similar data points, such as the time required to drive between healthcare service providers. [9]

Transport times were requested from both ground and air ambulance organizations, however this information was not made available.

Time of death was defined as the time of brain death declaration or cardiac death, whichever came first. If a neurosurgical procedure and another procedure were performed simultaneously (e.g. craniotomy and exploratory laparotomy), both times were recorded. Surgical start time was determined from the electronic anesthesia record as the time the patient arrived in operating room.

### Ethics

This study was approved by the institutional review board of the Massachusetts General Hospital (protocol 2010-P-002082/1). The need for informed consent was waived by this review board as the study involved materials (data, documents, & records) that were already collected and there was no interaction with human subjects. The IRB specifically considered (i) the risks and anticipated benefits, if any, to subjects; (ii) the selection of subjects, and (iii) the privacy of subjects and confidentiality of the data.

## Results

**Figure 1:**
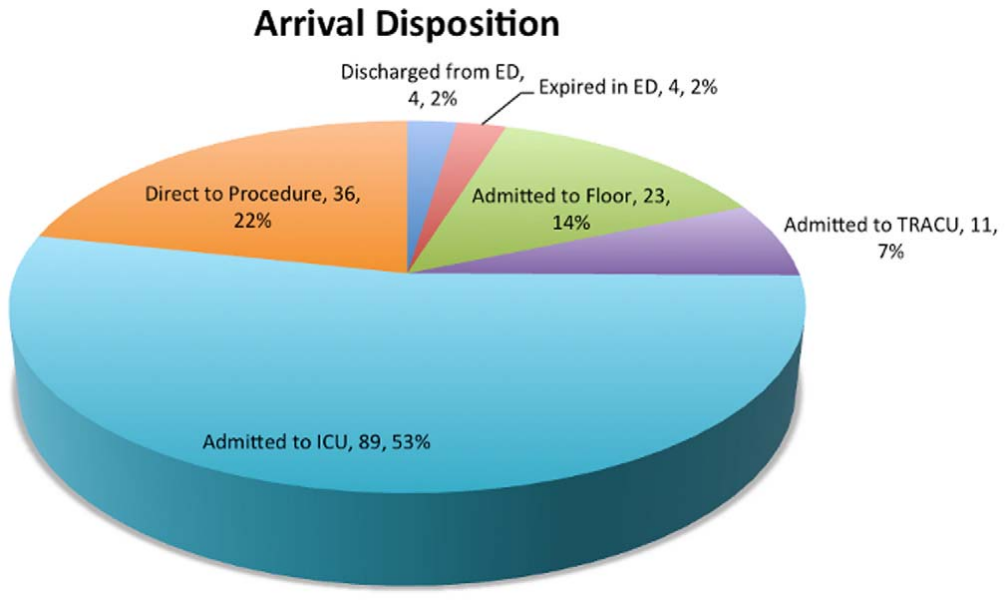
Disposition of patients following interfacility helicopter transport. The disposition of patients following helicopter transport varied among several locations in the hospital. A large proportion of patients were admitted to a non-ICU setting.

Of the 526 helicopter ambulance transports to our institution in 2008, we identified 167 meeting study criteria. All patients underwent one-way helicopter transport, though one patient was transferred a second time within the same week (and from the same referring hospital) for an unrelated condition. Mean age was 55.2, and 95 (57%) of the patients were male. Following arrival in the emergency department, four patients (2%) died, four (2%) were discharged directly from the emergency department, and the rest were admitted. One hundred twenty five (75%) patients were initially triaged to an ICU or taken directly to the operating room (Figure 1).

**Figure 2:**
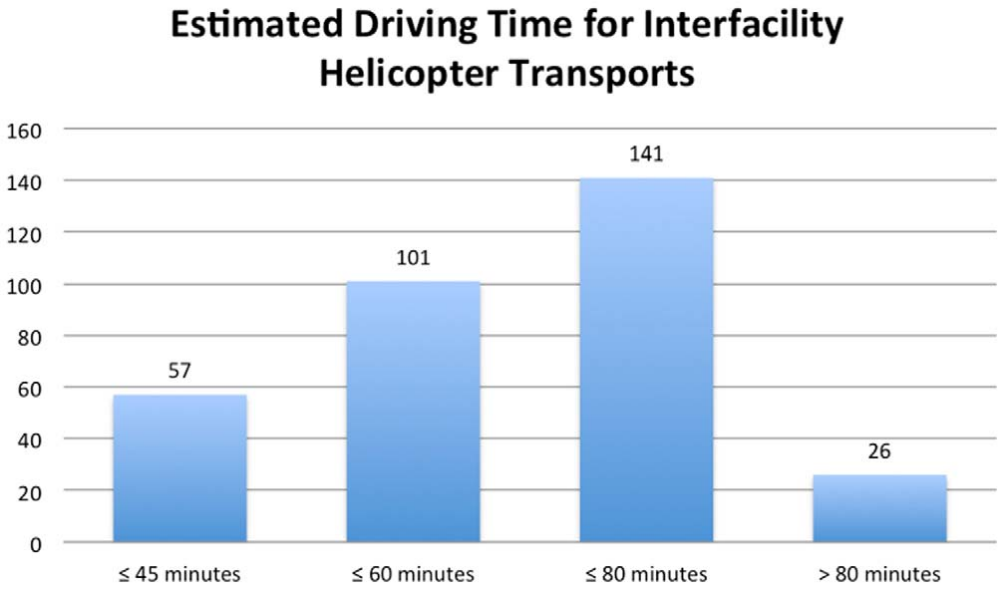
Estimated ground driving time for patients undergoing interfacility helicopter transport. Estimated driving times were then calculated by using Google Maps software (Google Inc. Mountain View, CA USA), using exact street addresses of door-to-door emergency department transport. The majority of patients transported were estimated ≤80 minutes.

The referring facilities were all located in the northeastern United States. Fifty seven patients (34%) were transported via helicopter despite an estimated ground driving time of ≤45 minutes (Figure 2). Only 26 patients (16%) arrived from facilities from which estimated ground driving times were >80 minutes.

Overall, 89 patients (53%) underwent at least one invasive procedure. Fourteen patients (8%) had invasive interventions performed either prior to or at the same time of a neurosurgical intervention, including emergent cricothyroidotomy, thoracotomy, and exploratory lapartomy. The median time to non-neurosurgical intervention in these patients was 29 minutes. Four of these patients also underwent neurosurgical intervention during the same admission, with a median time to neurosurgical intervention of 12.0 hours.

**Figure 3:**
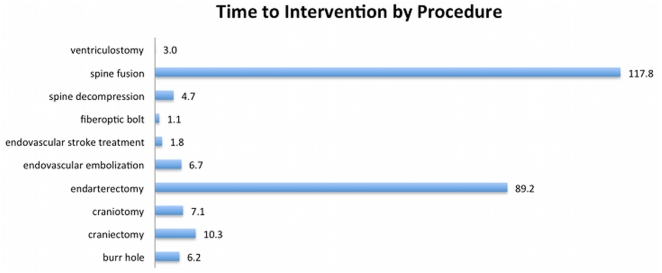
Median time to procedure by procedure. The median times to intervention are presented (hours). Fiberoptic intracranial pressure monitors had the shortest interval at 1.0 hours, whereas spine fusions were 117.8 hours.

Of patients who only underwent neurosurigcal intervention, the median time to the first procedure was 3.2 hours and varied widely with diagnosis and procedure performed (Figure 3). Fiberoptic intraparenchymal pressure monitors (fiberoptic bolts) in the setting of traumatic brain injury represented the shortest interval to intervention (median 1.01hours, n = 8) while spine fusion carried longest interval to intervention (median 117.8 hours, n = 2).

**Figure 4:**
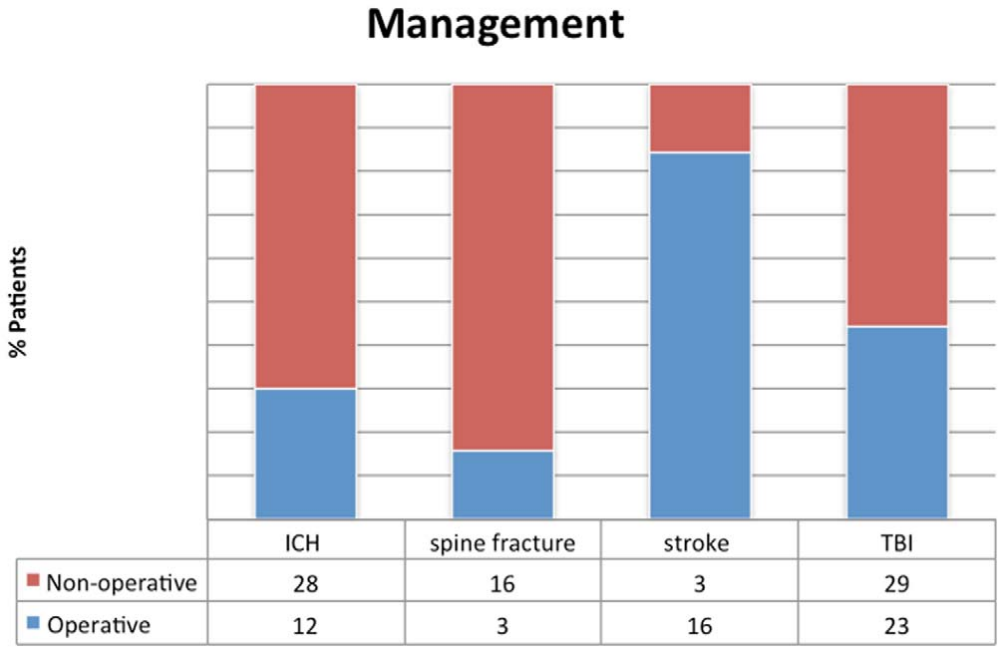
Operative versus non-operative management for selected diagnoses. The distribution of management, dichotomized as “operative” or “non-operative” for selected diagnosis. The majority of spine fractures had no invasive intervention, whereas the majority of stroke patients underwent endovascular stroke therapy.

Diagnoses for the study population included brain tumor, central nervous system infection, intraparenchymal hemorrhage, incidental cerebrovascular lesion, ruptured intracranial aneurysm, ruptured arteriovenous malformation, cerebrospinal fluid shunt malfunction, skull fracture, spinal cord injury, spine fracture, ischemic stroke, suspected traumatic brain injury, and traumatic brain injury (Figure 4).

**Figure 5:**
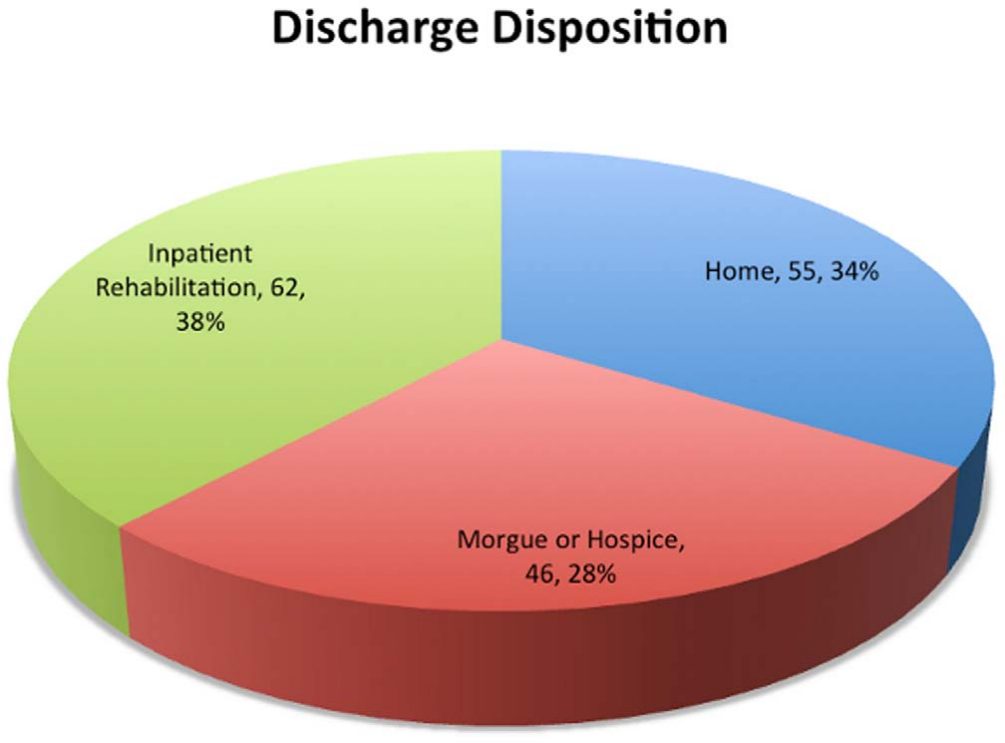
Discharge disposition for admitted patients following interfacility helicopter transport. Patients that were admitted to the hospital were ultimately discharged to home, inpatient rehabilitation, or the morgue/hospice.

Discharge disposition was inpatient rehabilitation for 62 patients (38%), home for 55 (34%), and death or hospice care in the remaining 46 (28%) (Figure 5). Six admitted patients (4%) were discharged home within 24 hours.

## Discussion

In an era of healthcare spending reform and scrutiny, the patterns in the mode of transportation for patient transport must be examined. Even though there is no national requirement to track or report fees associated with patient transport, helicopter costs can range from less than $12,000 to as much as $25,000 per flight, whereas ground ambulances are generally much less expensive, with an average transport to an emergency department estimated at $800 to $2,000.[10]

Interfacility transfer is now common for a variety of neurosurgical emergencies, and necessity for a higher level of care is the commonly cited justification.[3] [2] [11] Our observational analysis demonstrates that many patients transferred via helicopter ambulance for neurosurgical care indeed underwent an invasive intervention at our institution—an intervention which presumably was unavailable at the referring facility. And though not addressed explicitly here, many of these patients benefited from non-invasive measures (e.g. high-level ICU care) that may have been unavailable at the referring facilities. For example, there is evidence that strict blood pressure control can limit intraparencyhmal hematoma expansion. [12] This can be accomplished effectively in a dedicated neuro-critical care unit where outcomes have proven to be better in both aneurysmal subarachnoid hemorrhage and stroke patients. [13]–[14]

In initiating a helicopter ambulance transfer we assume that the referring practitioner held the following assumptions: (**1**) *this patient suffers from an acute neurosurgical condition requiring care at a higher-level facility*, (**2**) *helicopter transfer is more rapid than ground transport, and *(**3**) *the expected difference in time between ground and helicopter transport will be clinically meaningful.* As helicopter transport is costly, with estimates of $30,365 and $91,478 per beneficial mission for non-specific patient populations, each of these assumptions merits examination.[15]

With regards to assumption (**1**), numerous studies have indicated that the transfer of certain neurosurgical patients to high-volume academic facilities improves outcomes. In one analysis of patients with severe head injuries, transfer from a rural facility to a level I trauma center was associated with improved survival versus transfer to a level II center. [16] A similar finding holds for patients harboring intracranial aneurysms, with surgeon caseload and experience correlating with improved outcome. [17]–[18]

Yet, there are certain “less critical” neurosurgical diagnoses for which there is no evidence that transfer to a level I facility improves outcome. Mild traumatic brain injury, for example, can be managed effectively when a surgical lesion is not initially present. [19] In fact, some suggest that the availability of a neurosurgeon is not essential for managing mild traumatic brain injury if a properly trained and credentialed trauma surgeon or other health care provider can appropriately monitor a patient's neurologic status. [20] Telemedicine has also proven an effective adjunct in managing mild traumatic brain injuries, obviating the need for transfer in many cases. [21]

On the other end of the spectrum, patients with “highly critical” diagnoses, such as acute epidural hematoma, may not be suitable candidates for interfacility transfer. Any delay in intervention in such patients—even if to facilitate a higher level of care—may come at the expense of clinical outcome. Patients with epidural hematomas transferred for surgery have been shown to have poor outcomes [22]–[23], although craniotomy for epidural hematoma by non-neurosurgeons has also been associated with poor outcome. [24] Though the majority of referring hospitals in our study do not have continuous neurosurgical coverage, many of them have affiliated credentialed neurosurgeons. Intervention by these specialists in the setting of life-threatening situations may in many cases be preferable to interfacility transfer and its attendant delays in treatment.

Additionally, some patients will not benefit from any treatment or transport, such as those with massive intraparenchymal hematomas. For example, several patients in this study were transferred to our facility with >150 cc^3^ intracerebral hemorrhages for “neurosurgical care”, departing from the referring facility with an absence of brainstem reflexes. Five such patients were designated CMO in the emergency department upon arrival. Application of stroke scoring tools may help to facilitate family discussions at the referring hospitals regarding prognosis in these very grim situations, perhaps avoiding unnecessary transfer.[25]

Addressing assumption (2), evidence for any substantial difference in transport times for helicopter versus ground ambulance is lacking. Without documentation of the times of dispatch request, patient pick-up, patient arrival, and duration out-of-hospital, direct comparisons are a challenge. We used ground distance and a publicly available web-based route calculator to estimate ground driving times. Most patients in this study (60%) were transferred from a facility less than one hour away by ground. Helicopter transfer time is comprised not only of flight time (but also dispatch time, etc.), and as ground transport times fall below one hour, it is unclear that a helicopter could provide any time advantage over a ground ambulance. In one study, helicopter transport was faster than ground transport for interfacility transfer of patients from all hospitals studied in a regional referral system, however the time difference was miniscule. [26] In another study using historical controls, a hospital system that removed a hospital based air ambulance service did not demonstrate increased transport time or mortality for trauma patients.[27] It should be noted that while helicopter transport may or may not be faster than ground transport overall, there is evidence that ground dispatch times and “set up” times are shorter for ground transportation. [26]

Finally, even if helicopters reduce transfer times, it is not clear that this consistently confers a clinical benefit (assumption (**3**)). In select conditions, there is evidence that intervening within a narrow window is critical to good outcome. In addition to extra-axial hematomas (discussed above), beneficial effects of early treatment are seen in ischemic stroke.[28] Intravenous thrombolysis improves outcome in patients with ischemic stroke if given within the first 3 hours, and this window extends to 4.5-hours in select patients. [29]–[30] There is also compelling evidence that mechanical embolectomy can further extend this window, and endovascular therapy should therefore be considered in patients who fail or have contraindications for intravenous thrombolysis, or who present within 4.5–8 hours (and perhaps up to 12–24 hours for basilar occlusions). [31] The adage “time is brain” certainly holds for this population, and expedited transfer to a center with capability for such procedures may confer a clinical advantage.

While early treatment of ischemic stroke has proven benefit, the same is not true for several of the more common diagnoses in the cohort of patients under consideration here. For example, many spine fractures in this study did not require surgical intervention. And when surgery was performed, it was rarely done acutely. In such cases, there appears to be little (if any) clinical justification for the reduction in transfer time provided by costly helicopter transport. Furthermore, the median times to intervention (Figure 3) were lengthy enough that even if modest reductions in transfer time facilitated by helicopter were assumed, they probably did not benefit many patients.

In our study, 106 (63%) of patients either required no intervention at all or only underwent either fiberoptic bolt placement or ventriculostomy. In many cases, these patients could thus have been safely stabilized at the referring hospital or undergone ground transfer. Even if fiberoptic bolts and ventriculostomies are designated procedures necessitating urgent helicopter transfer (n = 8 and 20, respectively), this leaves 56 patients (34%) who ultimately did not require any invasive procedure and did not expire in the accepting facility. Additional study may continue to highlight those neurosurgical conditions that are most likely to benefit early treatment and establish the efficacy of interfacility helicopter transport.

### Limitations

Limitations of this study include the lack of detailed flight records, a control group of neurosurgical patients transported by ground ambulance, and weather and traffic conditions at the time of each transfer.

It is possible that estimations of ground ambulance transport time may be inaccurate during certain traffic conditions such as rush hour. It is also possible that ambulance transport could outperform a regular vehicle during non-rush hour traffic, with many of these driving time estimates potentially being overestimations. Without the actual data for exact times, any determination of transport time is merely estimation.

Interestingly, with regard to transport during rush hour (defined as arrival between 6–10 am and from 4–7 pm local time, only **28** patients fell into this category out of our series of 167 patients. We have previously demonstrated that for transfers received at our facility, patients are more likely to be transferred between midnight and 6 am (adjusted OR: 5.201; P = .000) compared with other time periods throughout the day. [3]
